# The Week's Work

**Published:** 1911-07-08

**Authors:** 


### The Week's Work.

### THE BRITISH HOSPITALS ASSOCIATION.

A memorial has been drawn up by the British Hospitals Association on behalf of the voluntary hospitals, for presentation to the Chancellor of the Exchequer. The committee chosen at the London Conference held at St. George's Hospital on June 2 has appointed a deputation to present the memorial, which consists of Sir William Cobbett, Sir Frederick Eve, Mr. Charles Lupton, Mr. Howard Collins, Mr. Conrad W. Thies, and Dr. Mackintosh, M.V.O. Our report of the Manchester meeting, which appears on another page, illustrates the interest which has been excited throughout the hospitals of the country as the result of the London Conference, and the text of the memorial is alluded to in it.

### MR. MORRIS ON THE INSURANCE BILL.

Mr. Ernest W. Morris, secretary of the London Hospital, has at length issued his opinion as to the probable effect of the Government's Insurance Bill upon the London Hospital. The result is interesting, because it illustrates by detailed examples the opinions that have been already expressed in The Hospital by hospital secretaries throughout the country. For instance, he does not merely tell us that this hospital will have to pay a large sum for the insurance of its own staff, but has worked that sum out at £856, which amount will give a rough indication of the new cost under this heading to other hospitals. The arguments showing the probability of the falling-off in the subscriptions of the large employer and of the middle-class and working-man are also pointed with similar detail, so that if there is little more fresh light to be shed on the question, the effect of the light we already have is heightened and made perspicuous. Mr. Morris writes excellently, as the readers of his book know, and this memorandum is exceedingly well put and forcible.

### THE NEW WING AT THE HOMŒOPATHIC HOSPITAL.

As we announced last week, the new Sir Henry Tyler extension wing at the London Homœopathic Hospital was opened last Wednesday by Princess Louise (Duchess of Argyll). Mr. Edwin T. Hall, F.R.I.B.A., of 54 Bedford Square, is the architect, and we hope to publish in an early issue the plans of this important addition to the hospital. We need only recall the fact that the foundation stone of the Tyler wing was laid on June 30, 1909, and that the wing itself consists of seven storeys. The lower ground floor has been added to the out-patient department. The ground floor has allowed the connection of the old board-room with Mr. Attwood's secretarial room and the Clerk's office, and the new board-room is a very handsome apartment. A new ward with eighteen beds, and a day room occupy the first floor, which is a model for the two floors above it, except that the third floor ward is devoted to children, and contains twenty-five beds, and an additional bath-room. On the fourth floor are ten small wards, with eleven beds for private patients, a small theatre unit, and a ward kitchen. The top or fifth floor contains four small wards with thirteen beds for children, surgical, or isolation cases. Finally, the roof is adapted for open-air treatment if required. To avoid misapprehension, it should be added that though the Sir Henry Tyler wing is the chief, or most prominent part of the extension, much reconstruction of the original part of the hospital has taken place. For instance, a new mortuary has been built, and, thanks to Dr. Margaret Tyler, the central of the three laboratories in connection with the pathological department has been fitted. These, with the new home, recently described in The Hospital, form a practically reconstructed hospital.

### THE "EAST SUSSEX" HOSPITAL.

The inverted commas in the heading of this paragraph indicate the new name of what used to be known as the Hastings, St. Leonards, and East Sussex Hospital. We have not yet been informed of the detailed reasons which have led the Board and Mr. J. W. Garnier, the hospital's secretary, to alter an unwieldy title into a common-sense, and, probably, colloquial one. On grounds of brevity alone the change is justified. The new name is better in every respect, and implies the service of a wider area than its more cumbersome and happily contracted predecessor.

### THE NEW PLANS FOR BRADFORD INFIRMARY.

We announced on June 24 that the first prize in the architects' competition for the best design of the proposed new Royal Infirmary at Bradford had been awarded to Mr. William A. Pite, F.R.I.B.A., 116 Jermyn Street, St. James's, S.W. We are now in a position to add that his general plan will be reproduced in The Hospital in an early issue. Our readers will hardly need reminding that Mr. Pite is the architect of the new King's College Hospital on Denmark Hill, the buildings of which were described in our issue of March 4 last. The hospital he has designed for Bradford will provide, we understand, accommodation for 382 beds, with possible additions to raise this number to 500. The general instructions issued to the competitors laid down the area of the site as twenty-four acres, stipulated for a two-storey pavilion system, and required the out-patient department and dispensary to be placed on the east, the boiler houses on the north-east, and the pathological and isolation buildings on the north of the land available. Detailed description, in fairness to Mr. Pite, must be postponed till it can be pointed by comparison with the actual architect's drawings, but, as will be expected, the general principle of "centralisation and radiation" has been aimed at.

### THE STOCKPORT INFIRMARY'S MEMORIAL.

Colonel A. J. Sykes, M.P., the Honorary Treasurer of the Stockport Infirmary, has been congratulated on an apparently unique success. In his double function as Hospital Treasurer and Mayor of Stockport, he urged the raising of

£10,000 for the extension of the Infirmary as a memorial to King Edward, and said how much he wished that this sum might be raised before the Coronation. He must, we think, not only have "expressed a wish," but have used his utmost influence for this purpose, since the sum, a record in amount and in the shortness of time in which it has been subscribed, was announced as raised during the Coronation festivities. "Never before," says the local Press, "has so much money been publicly subscribed in so short a time in Stockport." We have seen the apparently impossible in matters of hospital finance achieved so often to the surprise of inert committees that our experienced optimism in this respect hardly stands in need of the present example to strengthen it. But, while pointing the moral to all committeemen whom it may concern, we cannot resist congratulating Colonel Sykes, the Treasurer, and Major Tyler, the Secretary, on the success of their efforts.

### THE WORK OF THE YORK DISPENSARY.

The Maternity Hospital at York, which is really an extension of the York Dispensary, has just had a new ward dedicated by the Dean. We understand that about two and a half years ago the experiment was tried of extending the activities of the Dispensary by providing medical attendance for married women whose own homes are unsuitable for the occurrence of confinement, and of providing scope for the training of midwives, with the help of its outside maternity department. The work appears to have grown considerably, and the system of "baby consultations," which we noted on June 24 (page 316) as being so well carried on at the St. Marylebone General Dispensary in Welbeck Street, has been in force at York for eighteen months. The work done by such dispensaries is a recent but most valuable one, and one too that is bound to play a very much extended part in every city. Its pioneers, therefore, deserve the support of the public, and we hope that Mr. J. Peters, the secretary, and his committee have not been appealing in vain.

### A FOREIGN HOSPITAL RECORD.

A French sanatorium near Paris, on the east plateau of Bourg-la-Reine, is the Sanatorium de Larue, in the hamlet of Larue, a dependency of the Commune de Chevilly (Seine). In 1905 Mme. de Lauthonnye, with the help of a Committee of Patronage of which the Duchess de Brissac was president, founded a sanatorium for the treatment of tuberculosis and education in hygiene, with sixty-five beds wholly reserved for women and children. After a period of struggle and difficulty the charity is now in full swing. It has also received the support of the authorities, for the City of Paris and the Department of the Seine has founded thirty-four free medical beds in the institution, reserved for poor tuberculous women of Paris and the Seine Department. The rest of the patients have been until recently tuberculous women sent by private enterprise at a very moderate cost, and only cases capable of improvement have been received. It has now been decided to take in addition cases of surgical tuberculosis—men, women, and children—and a number of cases of joint and bone tuberculosis have been sent there by the Assistance Publique. This is a step in the right direction, for in many cases those who have been treated in the Paris hospitals for surgical tuberculous affections are unable to complete their cure owing to the lack of opportunity for rest in pure air amid sanitary surroundings. It is hoped that as the result of treatment in the sanatorium many cases will be prevented from relapsing which would otherwise almost inevitably have drifted back again to the hospitals for further operative treatment.

### THIS WEEK'S DRUG MARKET.

Business in the drug market has been more active during the week and a number of price changes, most of them in an upward direction, have taken place. Somewhat unexpectedly the price of opium has advanced materially and the market in Smyrna is rather excited; the advance is mainly due to the fact that the yield of the drug from the spring sown poppy plant is disappointing and it is expected that the yield will be considerably below the average. The advance is about 3s. per lb., but a further rise in value is probable. As a consequence makers of morphine have raised their quotations by 6d. per ounce, and makers of codeine have advanced their prices by 1s. 8d. per ounce, with the possibility of still higher prices. An addition has been made to the price of santonin notwithstanding the high figure already quoted. Carbolic acid is dearer with an advancing tendency. The price of balsam copaiba is well maintained. Balsam of tolu is dearer. Oil of savin, oil of wintergreen, and oil of theobroma are dearer. The price of oil of cloves has been slightly reduced. Gum acacia shows a tendency to advance in value. Cubebs and senega are dearer. The position of cod-liver oil is at present unchanged and the demand is exceedingly quiet.

